# Study of Quasispecies Complexity and Liver Damage Progression after Liver Transplantation in Hepatitis C Virus Infected Patients

**DOI:** 10.3390/genes12111731

**Published:** 2021-10-28

**Authors:** Meritxell Llorens-Revull, Josep Gregori, Cristina Dopazo, Francisco Rodriguez-Frías, Damir Garcia-Cehic, Maria Eugenia Soria, Qian Chen, Ariadna Rando, Celia Perales, Juan Ignacio Esteban, Josep Quer, Itxarone Bilbao

**Affiliations:** 1 Liver Diseases-Viral Hepatitis, Liver Unit, Vall d’Hebron Institut de Recerca (VHIR), Vall d’Hebron Hospital Universitari, Vall d’Hebron Barcelona Hospital Campus, Passeig Vall d’Hebron 119-129, 08035 Barcelona, Spain; meritxell.llorens@vhir.org (M.L.-R.); josep.gregori@gmail.com (J.G.); damir.garcia@vhir.org (D.G.-C.); maria.soria@vhir.org (M.E.S.); qian.chen@vhir.org (Q.C.); cperales@cbm.csic.es (C.P.); jiesteban@vhebron.net (J.I.E.); 2 Centro de Investigación Biomédica en Red de Enfermedades Hepáticas y Digestivas (CIBERehd), Instituto de Salud Carlos III, Av. Monforte de Lemos, 3-5, 28029 Madrid, Spain; cdopazo@vhebron.net (C.D.); frarodri@gmail.com (F.R.-F.); ibilbao@vhebron.net (I.B.); 3 Biochemistry, Molecular Biology, Universitat Autònoma de Barcelona (UAB), Campus de la UAB, Plaça Cívica, 08193 Bellaterra, Spain; 4 Roche Diagnostics SL, Avinguda de la Generalitat, 171-173, 08174 Sant Cugat del Vallès, Spain; 5 Hepatobiliopancreatic Surgery and Transplant Unit, Vall d’Hebron Institut de Recerca (VHIR), Vall d’Hebron Hospital Universitari, Vall d’Hebron Barcelona Hospital Campus, Passeig Vall d’Hebron 119-129, 08035 Barcelona, Spain; 6 Biochemistry and Microbiology Departments, Vall d’Hebron Institut de Recerca (VHIR), Vall d’Hebron Hospital Universitari, Vall d’Hebron Barcelona Hospital Campus, Passeig Vall d’Hebron 119-129, 08035 Barcelona, Spain; ariadna.rando@vhir.org; 7 Medicine, Universitat Autònoma de Barcelona (UAB), Campus de la UAB, Plaça Cívica, 08193 Bellaterra, Spain; 8 Surgery, Universitat Autònoma de Barcelona (UAB), Campus de la UAB, Plaça Cívica, 08193 Bellaterra, Spain

**Keywords:** hepatitis C virus, liver transplantation, fibrosis, variability, complexity measures, viral load

## Abstract

Cirrhosis derived from chronic hepatitis C virus (HCV) infection is still a common indication for liver transplantation (LT). Reinfection of the engrafted liver is universal in patients with detectable viral RNA at the time of transplant and causes fast progression of cirrhosis (within 5 years) in around one-third of these patients. To prevent damage to the liver graft, effective direct-acting antiviral (DAA) therapy is required as soon as possible. However, because of post-LT clinical instability, it is difficult to determine the optimal time to start DAAs with a low risk of complications. Evaluate changes in quasispecies complexity following LT and seek a predictive index of fast liver damage progression to determine the timing of DAA initiation. HCV genomes isolated from pre-LT and 15-day post-LT serum samples of ten patients, who underwent orthotopic LT, were quantified and sequenced using a next-generation sequencing platform. Sequence alignments, phylogenetic trees, quasispecies complexity measures, biostatistics analyses, adjusted R2 values, and analysis of variance (ANOVA) were carried out. Three different patterns of reinfection were observed (viral bottlenecking, conserved pre-LT population, and mixed populations), suggesting that bottlenecking or homogenization of the viral population is not a generalized effect after liver graft reinfection. None of the quasispecies complexity measures predicted the future degree of liver damage. Higher and more uniform viral load (VL) values were observed in all pre-LT samples, but values were more dispersed in post-LT samples. However, VL increased significantly from the pre-LT to 15-day post-LT samples in patients with advanced fibrosis at 1-year post-LT, suggesting that a VL increase on day 15 may be a predictor of fast liver fibrosis progression. HCV kinetics after LT differ between patients and are not fibrosis-dependent. Higher VL at day 15 post-LT versus pre-LT samples may predict fast liver fibrosis progression.

## 1. Introduction

Hepatitis C virus (HCV) infection is a cause of end-stage liver disease and an indication for liver transplantation (LT). The reinfection of the engrafted liver is universal in patients with detectable viral RNA during the first days, and even the first hours, after transplantation [1,2]. Symptomatic HCV hepatitis then develops in 1 to 4 months, although the clinical pattern varies. Within 5 years after LT occurs, there is a fast progression to cirrhosis in 10% to 30% of patients [3,4,5,6]. Before the introduction of interferon-free regimens, only 30% of non-transplant patients with HCV-related cirrhosis had liver decompensation at 10 years, whereas more than 40% of graft recipients showed decompensation within 12 months after the diagnosis of recurrent cirrhosis, and up to 60% experienced a decompensation episode 3 years later [4,7,8,9,10].

The factors that accelerate the post-LT progression of liver damage in HCV patients are uncertain and seem to depend on the characteristics of the virus and the patient [11,12]. Reports have shown that some amino acid signatures in the NS5B region of HCV are specific to patients developing cholestatic fibrosis hepatitis, which is a severe variant of HCV infection recurrence after liver transplantation [13]. However, there is little information on the diversity index and other viral factors that could help predict the accelerated progression of liver damage.

The advent of safe and highly effective direct-acting antiviral agents (DAAs) has profoundly changed the management of patients with advanced liver damage and those undergoing LT. Although DAA treatment before LT would be the best option, drug-to-drug interactions between some DAAs and various immunosuppressive agents may jeopardize this approach. Furthermore, some DAAs should be avoided in patients with severely impaired liver function and renal dysfunction because of complications, the most common being renal failure, which is not unusual after LT. An alternative is treatment soon after the procedure or when the risk of chronic rejection has decreased and immunosuppressive rejection medication is stable [14]. Hence, the question arises as to when would be the best time to start DAA treatment. The ELITA consensus statements [9] summarize the factors that should be considered to determine whether pre- and post-LT DAA therapy is justified in patients listed for decompensated cirrhosis without hepatocellular carcinoma (HCC). In general, patients who will progress faster to cirrhosis (fast progressors) should be treated earlier after LT than those with slower progression (slow progressors), who can be treated later, after the patient’s health status has improved. Therefore, finding a predictive index of fast liver damage progression to determine the timing of DAA initiation is of great interest, and quasispecies composition has been related to viral persistence, disease progression, and response to antiviral agents [15,16,17].

HCV is an enveloped, positive-sense, single-stranded RNA virus of the genus Hepacivirus in the Flaviviridae family [18]. The lack of proofreading activity of the nonstructural protein 5B (NS5B) RNA-dependent RNA polymerase leads to substantial sequence variation in the HCV genome, which displays mutation rates in the range of 10-3–10-5 mutations per nucleotide copied [19]. Hence, HCV does not exist as a single genome, even in a single infected individual; instead, it is a complex and dynamic distribution of non-identical but related genomes known as a viral quasispecies that undergoes a continuous process of genetic variation, competition, and selection [20,21,22,23,24,25]. The viral quasispecies complexity has been defined as the “intrinsic property that quantifies the diversity and frequency of haplotypes, independently of the population size that contains them” [26]. The relevance of the viral complexity level has been evidenced by the lower adaptability of viruses whose polymerase shows a higher or lower copying fidelity than the wild type with a comparable population size in the same biological context [27,28,29,30]. Therefore, when studying an infection, it is important to be aware that all members of the quasispecies may be relevant for establishing chronic infection, and an approach based on studying the diversity and complexity of the entire viral population should be considered.

The aim of this study was to evaluate changes occurring in the complexity of the HCV quasispecies soon after LT, seeking a parameter that would be predictive of fibrosis progression at 1 year following the procedure, which could be useful for guiding the timing of the start of DAA therapy.

## 2. Materials and Methods

This study consisted of ten HCV patients who underwent orthotopic LT, fulfilled the inclusion criteria, and had none of the exclusion criteria, as shown in the Supplementary Material.

This study was approved by the local institutional review board for clinical research, and all patients gave written informed consent in accordance with the 1975 Declaration of Helsinki.

Ten patients were included in the study, five being infected with HCV genotype 1 subtype a (G1a), four with G1b, and one with G3a [31]. Serum samples were collected 6 weeks before LT at the time they were included in the waiting list and 15 days after LT at the moment of hospital discharge.

The histological grade of liver fibrosis was evaluated according to the ISHAK fibrosis score [32] in liver biopsy specimens 1 year after transplantation. Viral load (VL) before LT (6 weeks) and 15 days after LT were measured using the Cobas 6800 system (Roche Applied Science, Basel, Switzerland; lower limit of detection, 10 IU/mL).

### 2.1. RNA Extraction, RT-PCR, Heminested-PCR Amplification and Quantification

A 30 µL amount of total RNA was obtained by extracting between 140 and 280 µL of the 6 weeks pre-LT and 15 days post-LT serum sample (depending on the VL of each) using the QIAamp Viral RNA Mini Kit 250 (Qiagen, Hilden, Germany) and following the manufacturer’s instructions. Samples P03 and P05 with the lowest VL required a double serum volume for RNA extraction to avoid bias occurring in the sequencing studies.

Reverse transcription polymerase chain reaction (RT-PCR) was performed as previously described [31]. Briefly, the reverse transcription of the NS5B region (nucleotides (nt) 8254–8707) was carried out using the Transcriptor One-Step reverse RT-PCR kit (Roche Applied Science, Basel, Switzerland). HCV RNA was reverse-transcribed into complementary deoxyribonucleic acid (cDNA) (30 min at 50 °C) and PCR-amplified for 35 cycles (10 s at 94 °C, 30 s at 53 °C, and 30 s at 68 °C) with specific oligonucleotides (5Bu8254: CNTAYGAYACCMGNTGYTTTGACTC; 5Bd8707: TTNGADGAGCADGATGTWATBAGCTC). A final product of 454 nt was obtained. Nucleotide positions were marked according to isolate H77 accession number AF009606 [33]. The reaction mixture for RT-PCR was prepared as follows: 28.5 µL H2O-PCR 1×, 10 µL buffer 5×, 2.5 µL DMSO, 1.5 µL upstream primer (20 pmol), 1.5 µL downstream primer (20 pmol), 1 µL polymerase, and 5 µL RNA.

Heminested PCR was then performed with 5 μL of DNA from the above PCR using the FastStart High Fidelity PCR System dNTPack kit (Roche Applied Science Basel, Switzerland) with 20 pmol of the labeled upstream primer (13N5Bo8254: GTTGTAAAACGACGGCCAGTCNTAYGAYACCMGNTGYTTTGACTC) and 20 pmol of the labeled downstream primer (13N5Bo8641: CACAGGAAACAGCTATGACCGARTAYCTGGTCATAGCNTCCGTGAA), both of which included a complementary universal M13 primer at the 5′ end. HCV DNA was amplified in a 35-cycle PCR (30 s at 94 °C, 30 s at 56 °C, and 30 s at 72 °C) with a reaction mixture comprised of 33 µL H_2_O-PCR 1×, 5 µL buffer 10×, 1 µL dNTP, 2.5 µL DMSO, 1.5 µL of 20 pmol upstream primer, 1.5 µL of 20 pmol downstream primer, 0.5 µL polymerase, and 5 µL DNA. A final product of 428 nt (including primers) was obtained.

PCR products from different isolates were pooled together before deep sequencing. Each isolate was tagged with a different multiplex identifier (MID) by performing a short (15 cycles) PCR. The final product was 498 nt in length (including primers, MID, and adaptors for GS–Junior sequencing), spanning nucleotides 8254–8641.

Amplification products were analyzed using 2% agarose gel electrophoresis with the QIAquick gel extraction kit (Qiagen, Valencia, CA, USA), quantified using the PicoGreen assay (Invitrogen, Carlsbad, CA, USA), and quality-analyzed using a BioAnalyzer DNA 1000 LabChip (Agilent, Santa Clara, CA, USA) prior to sequencing.

### 2.2. Ultradeep Pyrosequencing (UDPS)

Purified DNA from each sample was mixed, forming equimolar pools. Each pool was sequenced by UDPS-based next-generation sequencing (NGS) on the GS–Junior platform (454 Life Sciences, Roche, Branford, CT, USA), following the manufacturers’ protocol.

### 2.3. Nucleotide Haplotypes and Diversity Analyses

Sequencing data analysis was performed as previously reported [34,35]. Briefly, sequences were demultiplexed by the MID and specific primer and pairwise-aligned with respect to the dominant haplotype, excluding reads that did not cover the full amplicon or harbored more than 2 indeterminations or 3 gaps. Accepted indeterminations and gaps were repaired as per the dominant haplotype. Reads were collapsed to haplotypes and their corresponding frequencies. Only those with abundances above 0.1% and common to the forward and reverse strands were kept. All computations were made in the R language and platform [36] with in-house-developed scripts and with the help of the Biostrings [37], ape [38], seqinr [39], and ade4 [40] packages.

Diversity analyses were carried out on sequences that passed the filters, standardizing to the lowest coverage with a down-sampling and fringe-trimming approach [35]. The following diversity indices were used to define the viral quasispecies complexity at the molecular level [26]: mutation frequency (Mfmax), Hill numbers (qD) [41,42], and nucleotide diversity (π) [43,44].

### 2.4. Phylogenetic Analysis

For the phylogenetic analysis, read alignments were further filtered, discarding all haplotypes below 0.5% [35]. Haplotypes from the pre- and post-LT quasispecies were clustered by UPGMA (Unweighted Pair Group Method with Arithmetic mean) on the matrix of Kimura-80 genetic distances [45].

### 2.5. Biostatistic Analyses

Quasispecies complexity changes before and after LT were studied using the Mann–Whitney U test on each diversity index, with the null hypothesis of equal population means and the alternate hypothesis of greater diversity in pre-LT samples. Diversity indices were computed in the pre-LT and 15 day post-LT sample.

A principal component analysis was performed on the matrix of diversity measures, allowing the representation of samples characterized by variables that could be strongly correlated on orthogonal axes. The aim of this multidimensional exploratory analysis was to determine whether the diversity changes showed a general trend from pre-LT to post-LT when all indices were included in a multivariate analysis.

Associations between the liver damage level at 1 year post-LT and each diversity index in both the pre-LT and 15-day post-LT samples were studied by fitting a linear model of a single factor with two levels, high fibrosis (F3–F4) and low fibrosis (F0, F1, F2). The adjusted R2 was assessed as a measure of prediction accuracy.

Associations between the differences in VLs (log VL at 15 days post-LT minus log VL pre-LT) and liver damage level (high vs. low) at 1 year post-LT was carried out using a single fixed factor analysis of variance (ANOVA) with a significance level of 0.05.

The statistical methods used in this study were reviewed by Josep Gregori from Liver Diseases-Viral Hepatitis, Liver Unit, Vall d’Hebron Institut de Recerca (VHIR), Vall d’Hebron Hospital Universitari, Vall d’Hebron Barcelona Hospital Campus, Universitat Autònoma de Barcelona, Passeig Vall d’Hebron 119-129, Barcelona, Spain; Centro de Investigación Biomédica en Red de Enfermedades Hepáticas y Digestivas (CIBERehd), Instituto de Salud Carlos III, Madrid, Spain; Roche Diagnostics SL, Sant Cugat del Vallès, Barcelona, Spain.

## 3. Results

Patient data, viral load, and complexity measures are reported in Supplementary Table S1.

### 3.1. Viral Kinetics and Histological Grade

HCV RNA was detected in all patient samples: mean and standard deviation (SD) 1.52 × 10^6^ (SD 703492.881) at pre-LT and 2.91 × 10^6^ (SD 4655095.8) at 15 days post-LT. The following histological diagnoses according to the ISHAK classification were established in liver biopsies acquired at 1 year: F0 in 3 patients, F1 in 1, F2 in 2, F3 in 2, and F4 in 2 others. One of the two patients with F4 fibrosis grade (P10) developed cholestatic fibrosis hepatitis 6 months after liver transplantation.

### 3.2. Phylogenetic Studies

**Figure 1:**
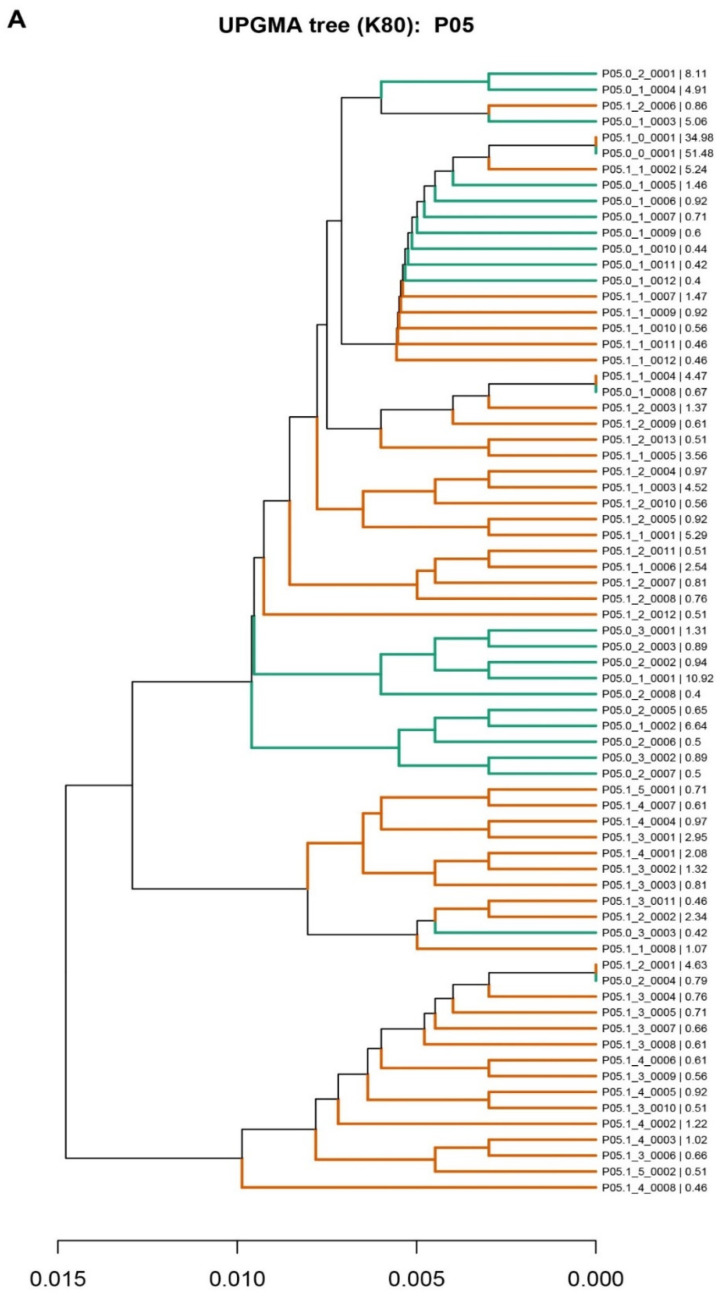

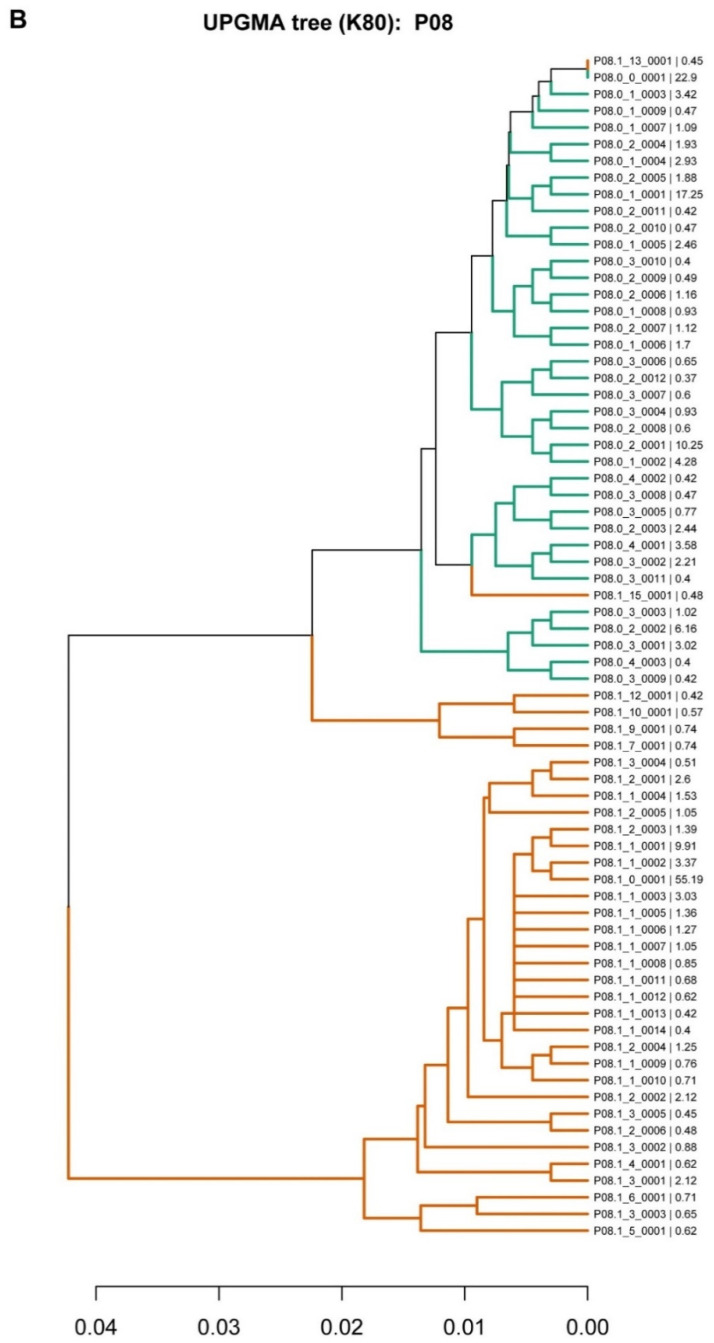

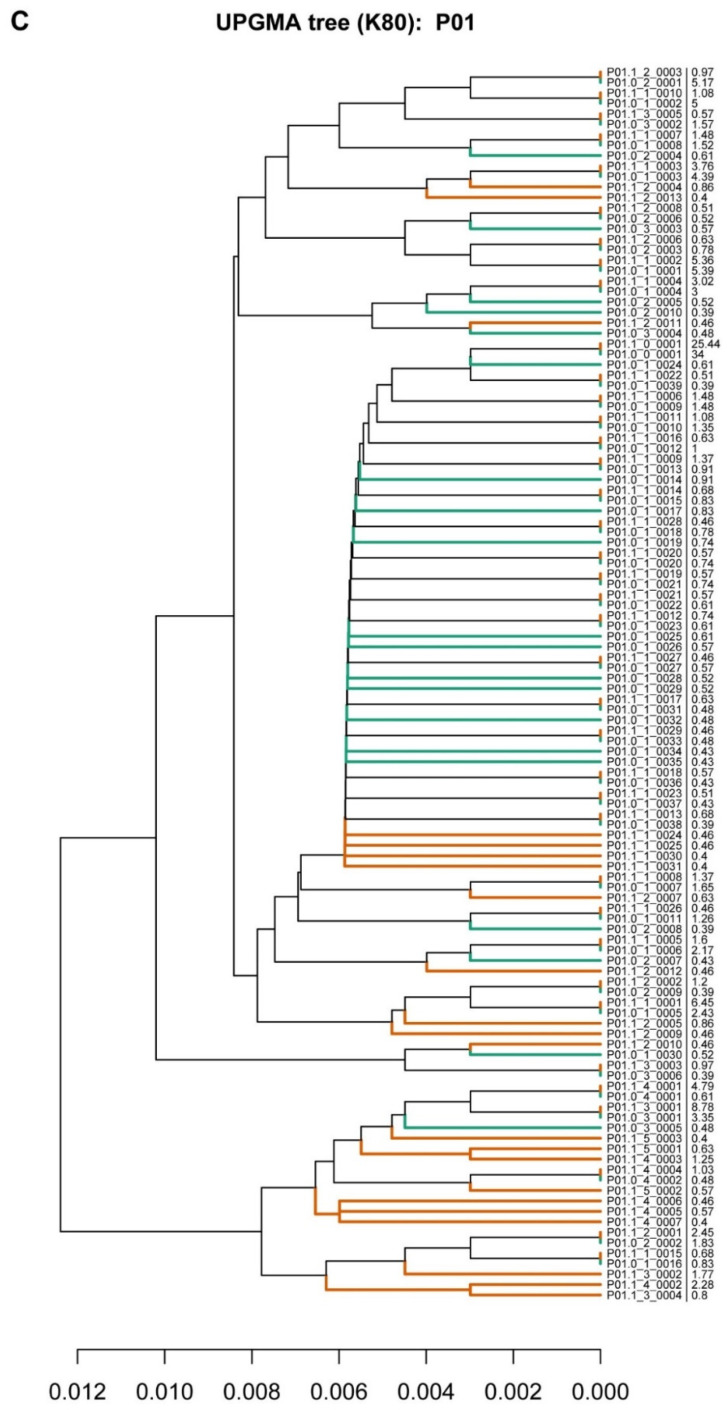
Phylogenetic trees representing the liver quasispecies population before (pre-LT) and after (post-LT) liver transplantation. Haplotypes from the pre-LT quasispecies are represented in green, and haplotypes from the post-LT quasispecies are in orange, as examples of the three infection patterns found. (**A**) Patient 05; (**B**) Patient 08; (**C**) Patient 01. The nomenclature used was Pxx.y_z_vvvv, where xx is the patient identifier (P01, P02 etc), y is the sample identifier (0 for pre-LT, 1 for post-LT), z is the number of differences between the haplotype and the master sequence, and vvvv is an identifier for haplotypes with the same number of mutations in the quasispecies. The last percent number represents the frequency at which each haplotype is represented in the quasispecies.

Three different patterns were obtained in the comparison of the phylogenetic analyses of pre- and post-LT samples (Figure 1). In the first pattern, observed in 7 of the 10 patients (70%), the master sequence (sequence with the highest percentage of reads) remained before and after LT. In this group, five of the patients were subtype 1a and two were subtype 1b. Interestingly, the master sequence was present in a higher percentage before than after LT in 4 of the 7 patients, remarking that 3 patients were HCV subtype 1a, while only 1 was 1b. In patient P10 who had a cholestatic event at six months after LT, the master sequence remained dominant after LT. In the analysis of the quasispecies in these patients, we found that most of the individual haplotypes were not common to both (pre- and post-LT) populations; instead, some specifically shared haplotypes had successfully developed new subpopulations (Figure 1A and Supplementary Figures S1–S6).

The second pattern, seen in 2 of the 10 patients, showed a clear viral “bottleneck” effect. The pre-LT master sequence had changed after LT, and two clearly separate quasispecies with no or only one shared haplotype were observed (Figure 1B and Supplementary Figures S1–S7). Both patients were HCV subtype 1b.

Finally, in the third pattern, observed in 1 of the 10 patients, the master sequence was highly conserved in both the pre- and post-LT quasispecies, with greater representation in the pre-LT samples. The vast majority of haplotypes were also conserved in the two populations and present in similar percentages (Figure 1C). The patient showed that the unique pattern corresponded with genotype 3a.

### 3.3. Unidimensional Analyses

For each diversity measure, a Mann–Whitney *U*-test was carried out, in which the null hypothesis was that diversity changes were random after transplantation, and, therefore, the average difference was 0. The alternative hypothesis was that diversity was reduced after transplantation due to the homogenization of the quasispecies, and the average difference was greater than 0.

**Figure 2:**
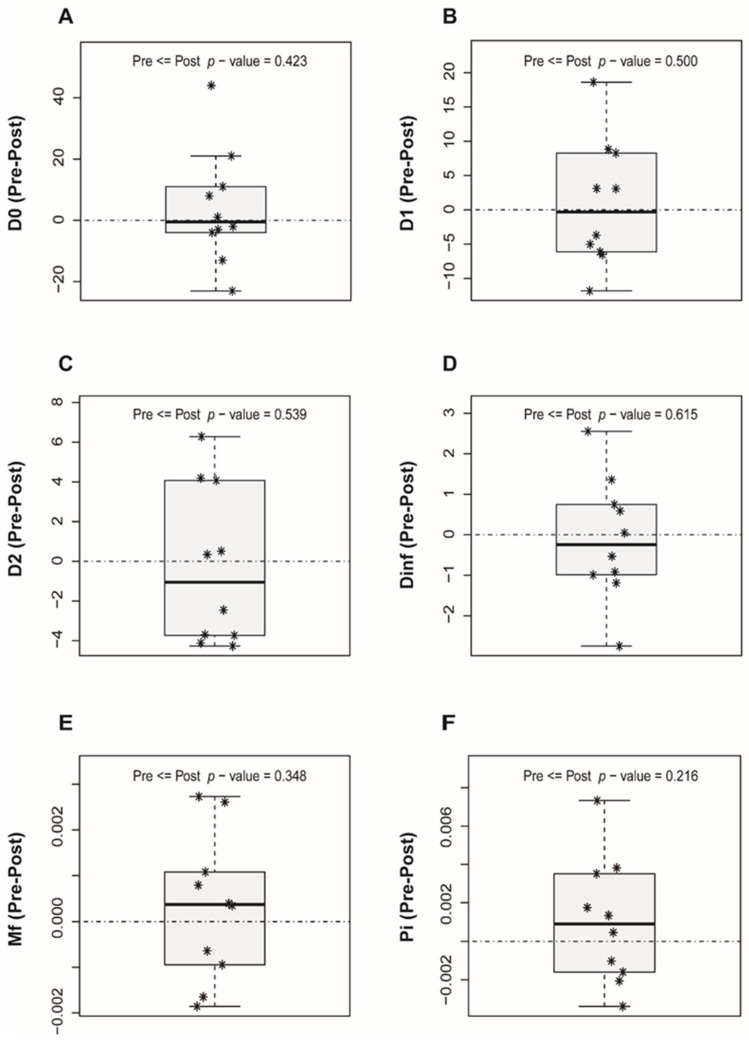
Boxplots with diversity indices showing differences between the pre-LT and post-LT quasispecies. (**A**) D0: hill number of order 0, (**B**) D1: hill number of order 1, (**C**) D2: hill number of order 2, (**D**) Dinf: hill number of order infinity, (**E**) Mfmax: mutation frequency, (**F**) π: nucleotide diversity. Each patient is represented as *. The *p*-value resulting from the Mann-Whitney *U*-test is included.

The differences (pre/post-LT) for each diversity measure with the resulting *p*-value from the Mann–Whitney *U*-test are shown in Figure 2A–F. In all cases, the 0 was well inside the observed distribution of differences and the *p*-values obtained were well above 0.05.

### 3.4. Principal Components Analysis

**Figure 3:**
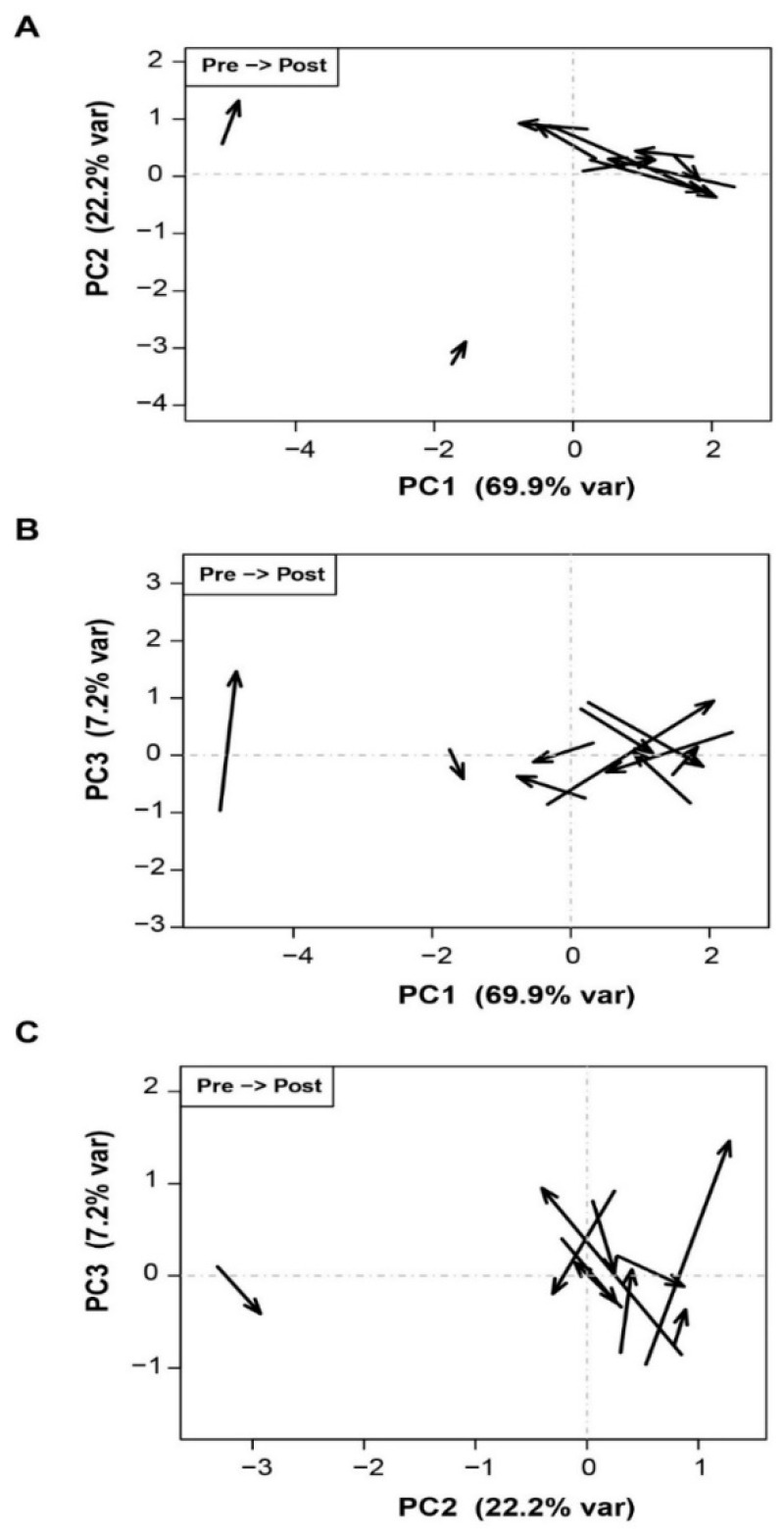
Samples represented on the planes of the first three principal components. Each pair of samples is represented as an arrow, with pre-LT at the tail and post-LT at the head. (**A**) PC2 vs. PC1; (**B**) PC3 vs. PC1; (**C**) PC3 vs. PC2.

As in the unidimensional analyses, changes towards lower viral complexity after transplantation were not confirmed. In Figure 3A–C, a significant multivariate association between LT and the diversity measures would result in a consistent pattern of arrows pointing in approximately the same direction, which is not the case.

### 3.5. Associations between Diversity Measures and Fibrosis at 1 Year Post-LT

Liver fibrosis as a measure of liver damage was dichotomized into low (F0, F1) and high (F3, F4) values. The distribution of values for the various diversity indices in each liver damage group and in pre-LT and 15 days post-LT samples is shown in Table 1. Here, the adjusted R2 value acts as an assessment of the prediction accuracy. Of note, the adjusted R2 was very low for all of the diversity measures, indicating that they lacked predictive capability in both samples.

### 3.6. Associations between VL and Fibrosis at 1 Year Post-LT

**Figure 4:**
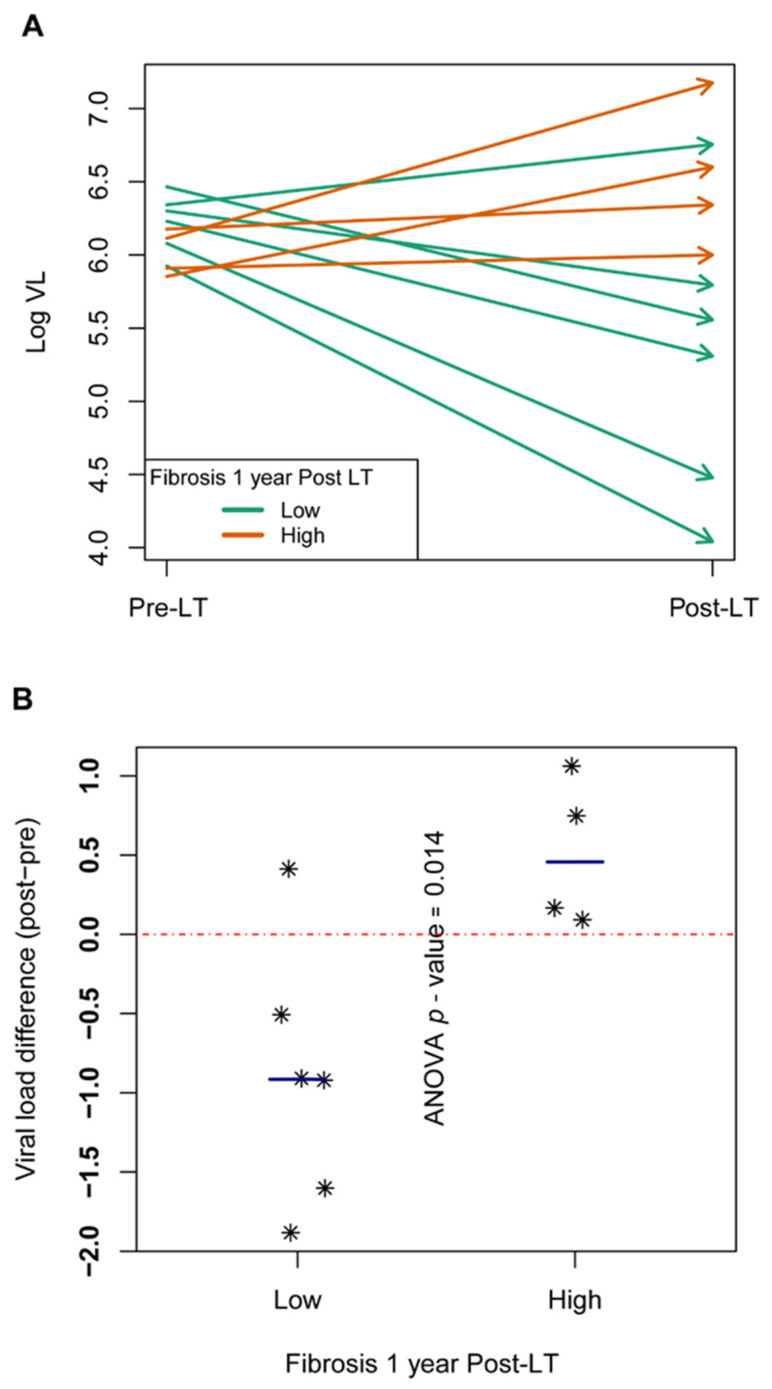
Viral load differences between pre and post-LT samples. (**A**) Arrows showing the evolution of log VL from pre-LT (arrow tails on the left) to 15-day post-LT (arrow heads on the right side of the box). At one year post-LT, patients with high fibrosis (F3–F4) in orange, patients with low fibrosis (F0–F2) in green. (**B**) Scatterplot with differences in log VL at 15 days post-LT minus pre-LT. The ANOVA *p*-value in the comparison of the two levels of liver damage is included. Each patient is represented as *.

Patients showing a VL increase at 15 days post-LT relative to the pre-LT value developed a higher degree of fibrosis (F3, F4) at 1 year post-LT; therefore, they could be considered fast progressors. In contrast, patients showing a VL decrease after LT could be considered slow progressors, as all showed minimal liver fibrosis changes (F0, F1) at 1 year after transplantation. The VL values were high and uniform in all pre-LT samples but were more dispersed in the post-LT samples (Figure 4A). In the ANOVA, a statistically significant association between VL and the progression of liver damage was found (*p* = 0.0144) (Figure 4B).

## 4. Discussion

Liver replacement causes a drastic change in the environment in which the virus had adapted to proliferate; therefore, changes would be expected to occur in the composition of the quasispecies following LT. After the primary HCV target organ is removed, there is a substantial reduction in the overall VL, and the viral populations remaining in the bloodstream and extra-hepatic reservoirs initiate infection of the new liver graft [1,46]. The number of viral particles (population size) starting the reinfection, together with differences in the liver graft, state of immunosuppression, and patient characteristics, may influence the evolutionary outcome and affect the quasispecies composition [22,47,48].

In this scenario, during graft reinfection, a genetic phenomenon known as “bottlenecking” is expected [49] in which the fittest virions are selected in the absence of an effective immune response, thereby increasing their frequency in serum with still limited variability [50]. In this sense, our phylogenetic studies have revealed three different patterns of viral behavior after liver graft infection. In the first pattern, observed in most patients (70%), the master sequence was conserved, but the mutant spectra differed. Only some sequences were the same as the pre-LT ones, suggesting that the reinfection of the liver was caused by the master and the most prevalent pre-LT sequences. In the two patients with the second pattern, the master sequence differed from the pre-LT one, and the mutant spectra were completely different, suggesting that reinfection of the liver graft was caused by a minor mutant from the pre-LT quasispecies. Finally, in the third pattern (one patient), the pre-LT quasispecies was maintained after liver transplantation, suggesting massive reinfection of the liver graft by most of the pre-LT virions. Despite pattern 2 and 3 only include patients with subtypes 1b and 3 respectively, the small sample size (two for pattern 2 and one for pattern 3), do not allow us to extract conclusions on the effect of the subtype in the viral quasispecies behavior after LT. However, pattern 1 has been found in patients with subtype 1a and 1b, suggesting that in most of the cases, and independently of the liver damage progression, HCV reinfection follows this particular pattern master sequence, which remains after LT but with different mutant spectra. Interestingly, in the patient that had a cholestatic fibosis hepatitis (P10) master sequence, it remained dominant after LT as previously reported by Gambato et al. in which 62% of cholestatic patients showed the remaining of master sequence compared with the 11% in the patients with mild recurrence [13]. The main limitation of this study is the small sample size. The main reason for the low number of patients included is that most of the patients are efficiently treated with DAAs before and soon after LT, thus limiting the number of samples fulfilling inclusion criteria for our study in which we require patients with at least one year of untreated follow-up.

It is widely recognized that liver fibrosis rapidly progresses in some patients, leading to cirrhosis at 1 year post-LT (fast-progressors), whereas others show minimal changes in the transplanted graft at the same time point (slow-progressors). In this situation, we hypothesized that the viral complexity indices might predict the fast or slow progression of liver damage. However, after conducting an exhaustive analysis of changes in the HCV quasispecies complexity measures (qD, Mfmax, and π) obtained before LT and 15 days following LT, none of the measures studied were significantly associated with progression to more aggressive liver damage at 1 year following the procedure. This may be because the viral quasispecies is a highly variable and dynamic population that fluctuates greatly over time, and the results may vary according to the moment at which the sample is collected and analyzed. Hence, we cannot exclude that the analysis of samples taken at a later point after LT might have led to the identification of a complexity index predictive of fast liver damage progression.

Interestingly, we observed a significant increase in VL values from pre-LT to 15 days post-LT in all patients who had an advanced stage of fibrosis 1 year after the procedure, in accordance with previous findings [51]. Our results support the notion that the difference in VL before and after LT may be of value in predicting fibrosis progression. Thus, VL changes may be a useful criterion to determine whether to administer DAA treatment as soon as possible post-LT or delay it until the patient is clinically stable.

## 5. Conclusions

To sum up, none of the viral complexity measures studied at 15 days after liver transplantation were significantly associated with liver damage progression at 1 year following the procedure. Three different patterns of liver graft reinfection were observed based on phylogenetic analyses. VL values were high and uniform in all pre-LT samples but were more dispersed in the post-LT samples. An increase in viral load after liver transplantation was associated with fast progression to liver fibrosis and could be an indicator that effective antiviral treatment should be started as soon as possible in these particular patients.

## Figures and Tables

**Table 1:** Ability of diversity measures (qD of order 0, 1, 2, infinity, Mfmax, and π) to predict high or low fibrosis degree at 1 year post-LT using both pre-LT and 15 day post-LT samples. Prediction accuracy of each diversity measure is represented as adjusted R2.

	Diversity Measures					
	Adjusted R^2^ (D0)	Adjusted R^2^ (D1)	Adjusted R^2^ (D2)	Adjusted R^2^ (Dinf)	Adjusted R^2^ (Mf)	Adjusted R^2^ (π)
Pre-LT	0.086	0.064	0.118	0.111	0.021	0.027
Post-LT	0.060	0.026	0.019	0.025	0.063	0.051

## Data Availability

The data presented in this study are available on request from the corresponding author.

## Supplementary Materials

The following are available online at https://www.mdpi.com/article/10.3390/genes12111731/s1, Figures S1–S7: Phylogenetic trees representing the liver quasispecies population before (pre-LT) and after (post-LT) liver transplantation. Table S1. Clinical data, viral load, and pre/post-LT viral complexity measures. Inclusion and exclusion criteria.
