# Supplementary material for: Study of Quasispecies Complexity and Liver Damage Progression after Liver Transplantation in Hepatitis C Virus Infected Patients
Source: Genes (Basel). 2021 Oct 28;12(11):1731. doi: 10.3390/genes12111731 (PMC8625210; doi:10.3390/genes12111731)
Supplement: Supplementary file 1 [file genes-12-01731-s001.zip › Llorens M et al Supplementary Material_Inclusion exlusion criteria.pdf]

Inclusion criteria

1. Recipient of the first orthotopic liver transplant

2. Adult of either sex

3. Age ≥18 years

4. Positive HCV RNA testing at 12 months before transplantation

5. Written informed consent for participation in the study

6. Able to participate in the study for the 12 months following transplantation

Exclusion criteria

1. Recipient of multiple organ transplants or a previous organ transplant

2. Split or living donor recipient infection

3. Recipient with ABO incompatibility

4. Recipient seropositive to HIV antibodies

5. Recipient due to fulminant liver failure

6. Recipient due to HCV infection under DAA treatment, with negative HCV RNA status

7. Known neoplasm or history of neoplastic disease, except basal cell skin carcinoma or hepatocarcinoma meeting the following criteria: no vascular invasion. Single nodule ≤5 cm diameter. or 2 or 3 three nodules <3 cm (Milan's criteria)

8. Glomerular filtration rate <60 ml/min/1.73m2 before transplantation or kidney dialysis requirement before transplantation

9. Critical illness with clinical instability that could affect the study goals

10. Patient treated with a new therapy under investigation within 1 month before transplantation with a drug that will be needed in the post-LT period

11. Patient with <103 viral particles 15 weeks post-LT
