# Supplementary material for: Study of Quasispecies Complexity and Liver Damage Progression after Liver Transplantation in Hepatitis C Virus Infected Patients
Source: Genes (Basel). 2021 Oct 28;12(11):1731. doi: 10.3390/genes12111731 (PMC8625210; doi:10.3390/genes12111731)
Supplement: Supplementary file 1 [file genes-12-01731-s001.zip › Llorens M et al Supplementary Material_table.pdf]

Table S1. Clinical data base, viral load and pre/post-LT viral complexity measures.

Patients	P01	P02	P03	P04	P05	P06	P07	P08	P09	P10
Subtype	3a	1a	1a	1b	1a	1b	1a	1b	1a	1b
Donor age	56	30	67	67	62	20	62	33	54	63
Receiver age	64	60	49	65	54	60	58	67	49	63
Liver damage[a]	low	low	low	low	low	low	high	high	high	high
ISHAK[a]	F0	F0	F0	F1	F2	F2	F3	F3	F4	F4
**Viral load**										
Pre-LT	2.00E+06	1.70E+06	8.40E+05	2.92E+06	1.20E+06	2.20E+06	1.50E+06	7.13E+05	1.30E+06	8.10E+05
Post-LT	6.22E+05	2.04E+05	1.10E+04	3.60E+05	3.00E+04	5.70E+06	2.20E+06	3.99E+06	1.50E+07	1.00E+06
**Number of different mutations**										
Pre-LT	39	49	44	42	19	38	33	12	28	22
Post-LT	35	19	22	11	22	24	25	33	37	26
**Number of polymorphic sites**										

**Pre-LT**	38	48	43	40	19	35	32	12	28	22
**Post-LT**	35	19	20	11	22	24	24	33	37	26
**Number of nucleotides sequenced**										
**Pre-LT**	772800	666288	1302000	944496	1614816	1276800	1734768	1445136	456960	1149120
**Post-LT**	589008	910560	1426992	1947456	660912	1274448	1465296	1187088	1036896	898800
**Number of haplotypes**										
**Pre-LT**	58	89	49	62	24	43	42	36	42	36
**Post-LT**	60	68	41	18	47	47	31	35	55	39
**Abundance of dominant haplotype**										
**Pre-LT**	34.00	17.40	57.24	31.52	51.48	25.66	45.61	22.90	29.34	24.59
**Post-LT**	25.44	11.77	34.05	38.70	34.98	31.74	46.57	55.19	25.37	36.93
**MF min. minimum mutation frequency**										
**Pre-LT**	5.05E-05	7.35E-05	3.38E-05	4.45E-05	1.18E-05	2.98E-05	1.90E-05	8.30E-06	6.13E-05	1.91E-05
**Post-LT**	5.94E-05	2.09E-05	1.54E-05	5.65E-06	3.33E-05	1.88E-05	1.71E-05	2.78E-05	3.57E-05	2.89E-05
**MF max. maximum mutation frequency**										
**Pre-LT**	2.80E-03	1.50E-02	1.57E-03	3.50E-03	1.98E-03	2.38E-02	1.86E-03	4.15E-03	4.06E-03	5.27E-03

**Post-LT**	4.45E-03	1.22E-02	2.52E-03	3.16E-03	3.84E-03	2.30E-02	2.50E-03	3.07E-03	3.67E-03	2.66E-03
**Mfe. mutation frequency per entity**										
**Pre-LT**	4.36E-03	1.62E-02	3.52E-03	6.24E-03	4.59E-03	2.80E-02	3.54E-03	6.45E-03	5.60E-03	7.52E-03
**Post-LT**	5.70E-03	1.32E-02	4.65E-03	5.46E-03	7.16E-03	2.76E-02	4.42E-03	1.04E-02	5.47E-03	4.20E-03
**^∏. Nucleotide diversity**										
**Pre-LT**	4.99E-03	2.19E-02	3.02E-03	6.54E-03	3.45E-03	3.04E-02	3.59E-03	7.07E-03	7.09E-03	8.32E-03
**Post-LT**	7.06E-03	1.45E-02	4.63E-03	4.80E-03	6.84E-03	2.69E-02	4.62E-03	5.72E-03	6.63E-03	4.51E-03
**^∏e. Nucleotide diversity by entity**										
**Pre-LT**	7.98E-03	2.40E-02	6.85E-03	1.12E-02	8.10E-03	3.29E-02	6.91E-03	1.04E-02	1.00E-02	1.19E-02
**Post-LT**	9.52E-03	1.55E-02	8.44E-03	8.03E-03	1.17E-02	2.85E-02	8.24E-03	1.77E-02	1.01E-02	7.70E-03
**D1. Hill numbers**										
**Pre-LT**	21.39	52.84	9.43	25.83	6.65	16.62	13.08	16.25	18.86	17.07
**Post-LT**	26.40	43.99	15.95	7.19	18.45	20.32	9.99	7.97	24.99	13.94
**D2. Hill numbers**										
**Pre-LT**	7.58	22.62	2.99	8.85	3.40	8.75	4.55	9.41	8.80	10.05
**Post-LT**	11.28	26.89	7.11	4.66	7.13	8.24	4.21	3.13	11.26	5.98
**D∞. Hill numbers**										

**Pre-LT**	2.94	5.75	1.75	3.17	1.94	3.90	2.19	4.37	3.41	4.07
**Post-LT**	3.93	8.50	2.94	2.58	2.86	3.15	2.15	1.81	3.94	2.71

[a] *Data from post-LT samples.*
