# Supplementary figures and images for: Study of Quasispecies Complexity and Liver Damage Progression after Liver Transplantation in Hepatitis C Virus Infected Patients
Source: Genes (Basel). 2021 Oct 28;12(11):1731. doi: 10.3390/genes12111731 (PMC8625210; doi:10.3390/genes12111731)

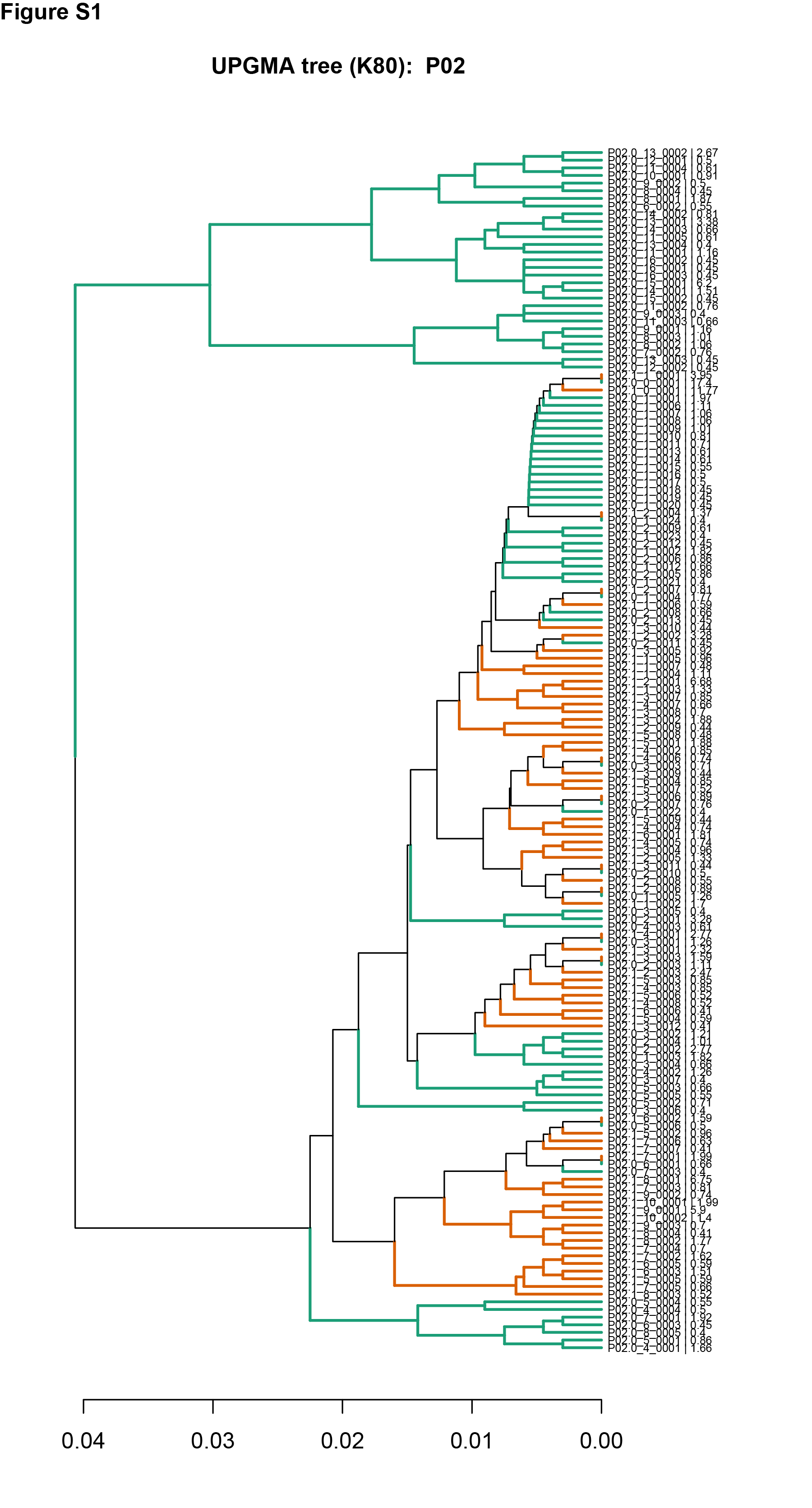

Supplement: Supplementary file 1 [file genes-12-01731-s001.zip › HplTreeS1.1.tif]

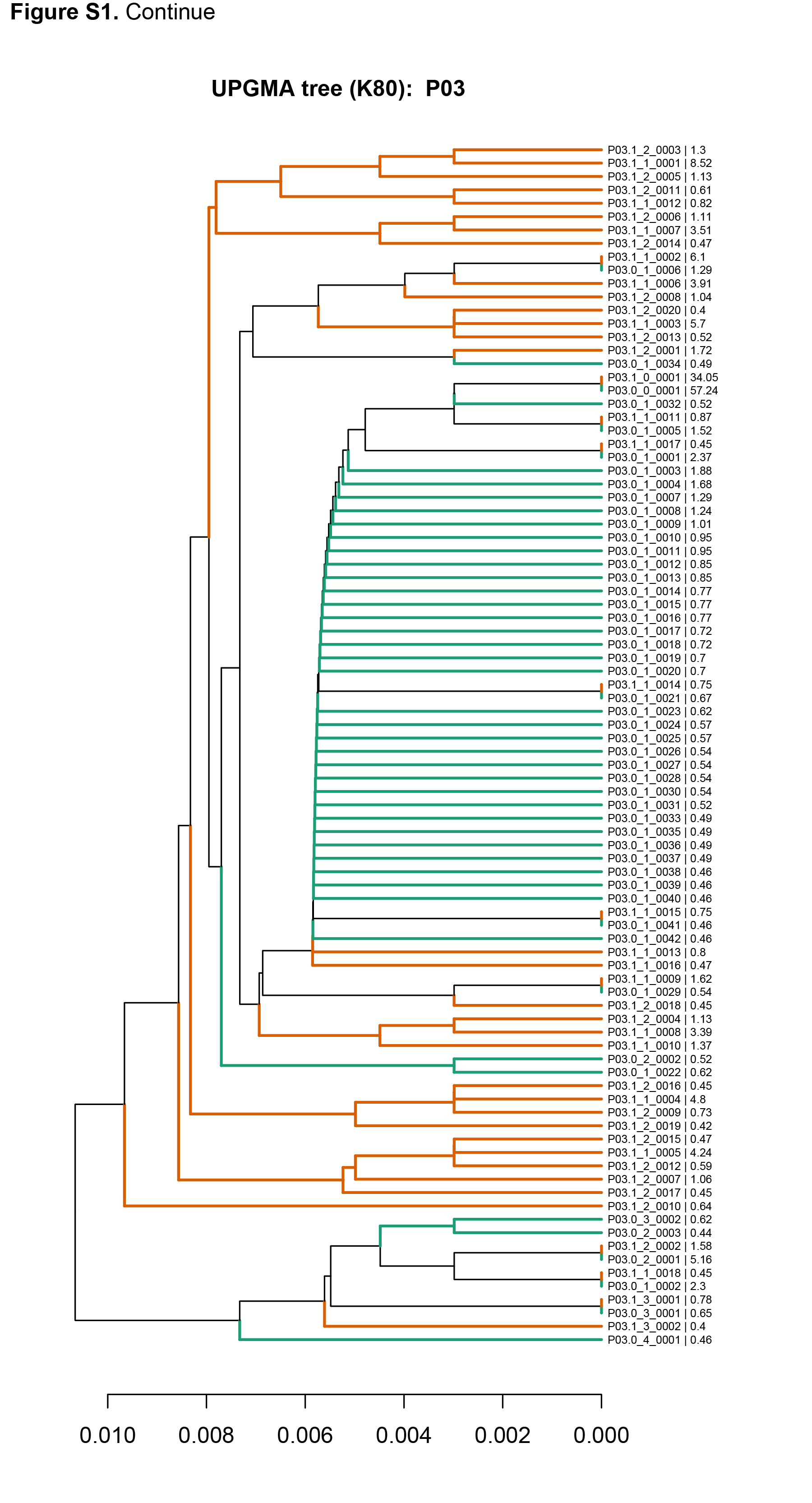

Supplement: Supplementary file 1 [file genes-12-01731-s001.zip › HplTreeS1.2.tif]

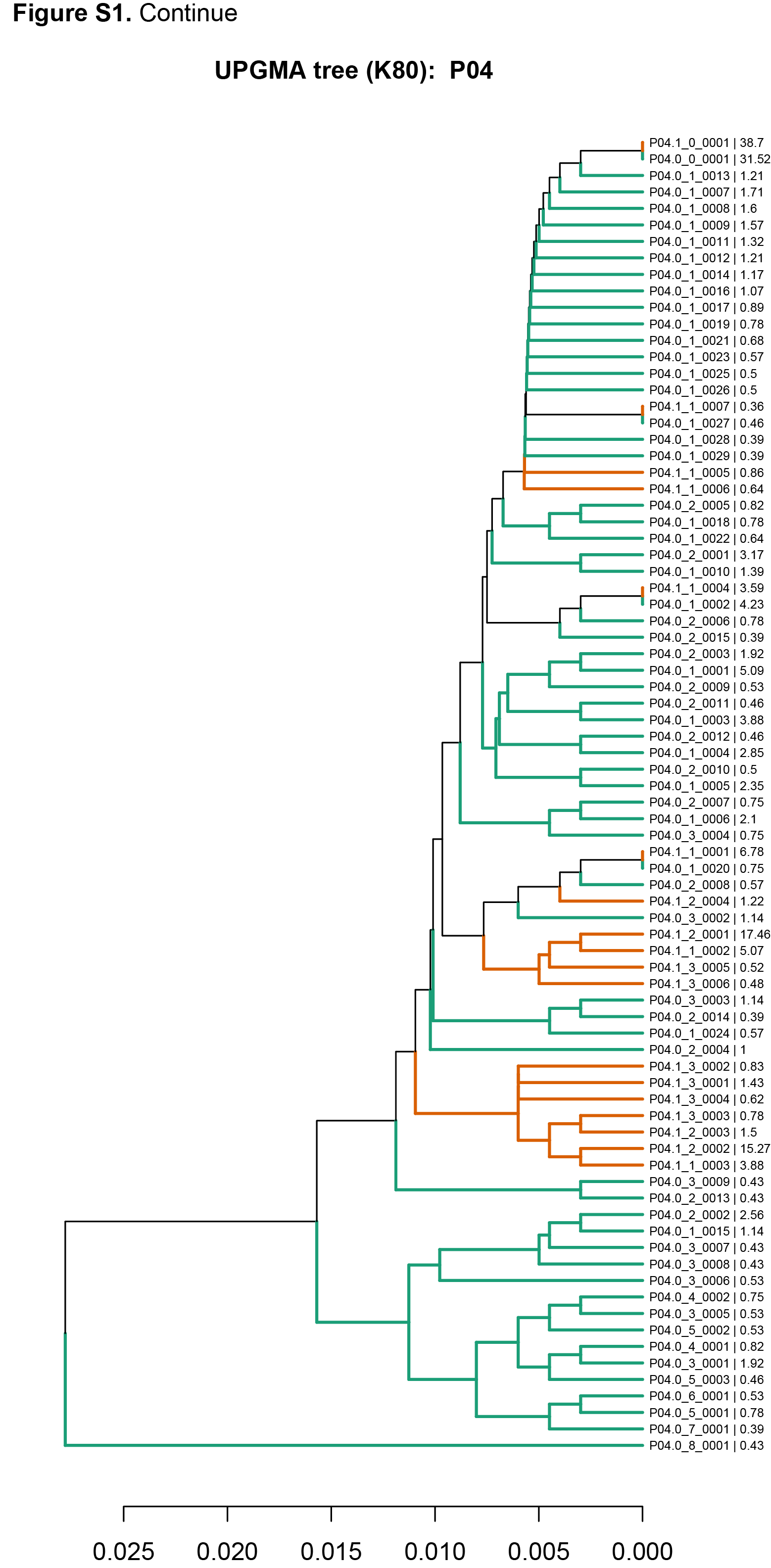

Supplement: Supplementary file 1 [file genes-12-01731-s001.zip › HplTreeS1.3.tif]

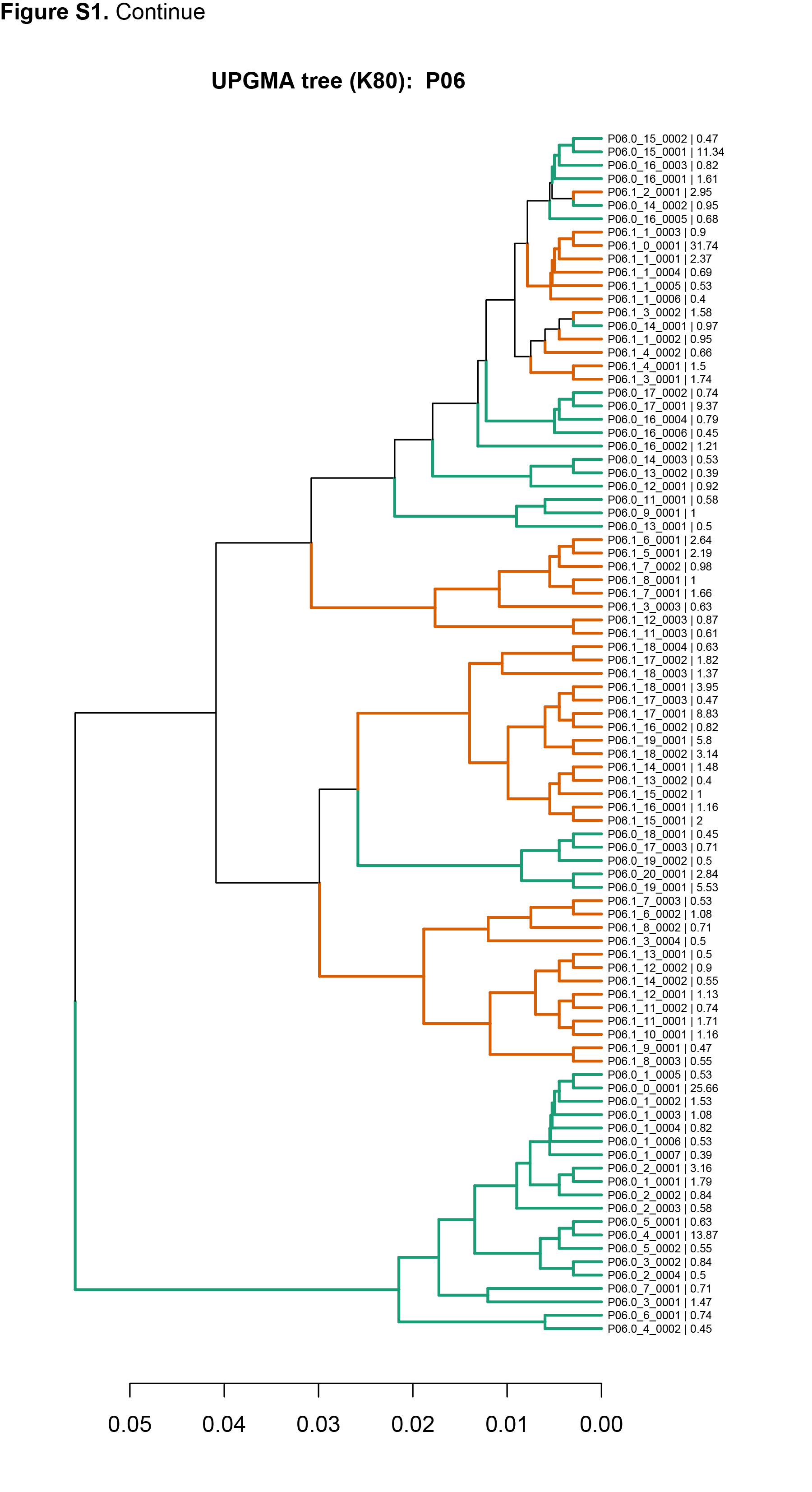

Supplement: Supplementary file 1 [file genes-12-01731-s001.zip › HplTreeS1.4.tif]

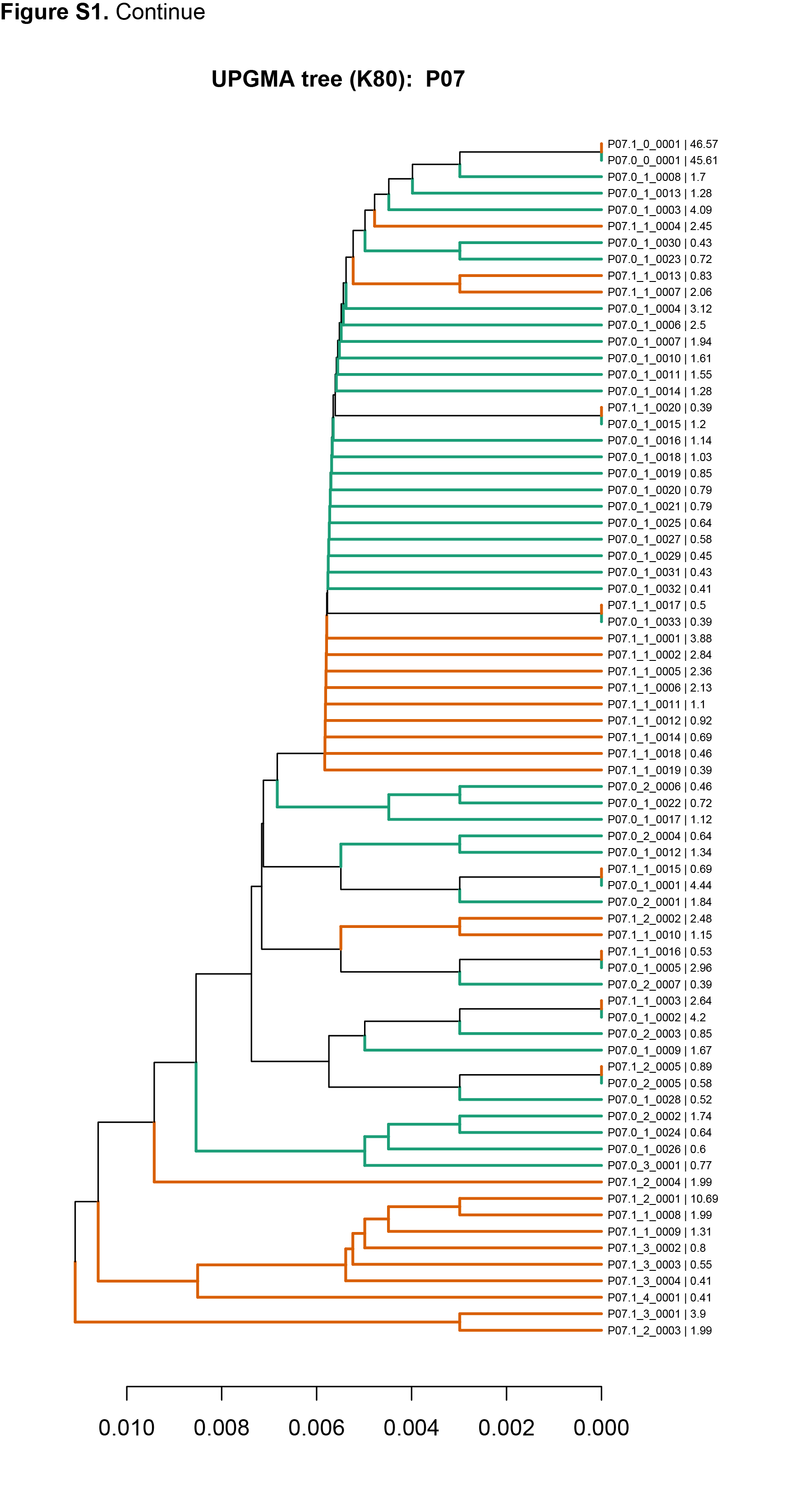

Supplement: Supplementary file 1 [file genes-12-01731-s001.zip › HplTreeS1.5.tif]

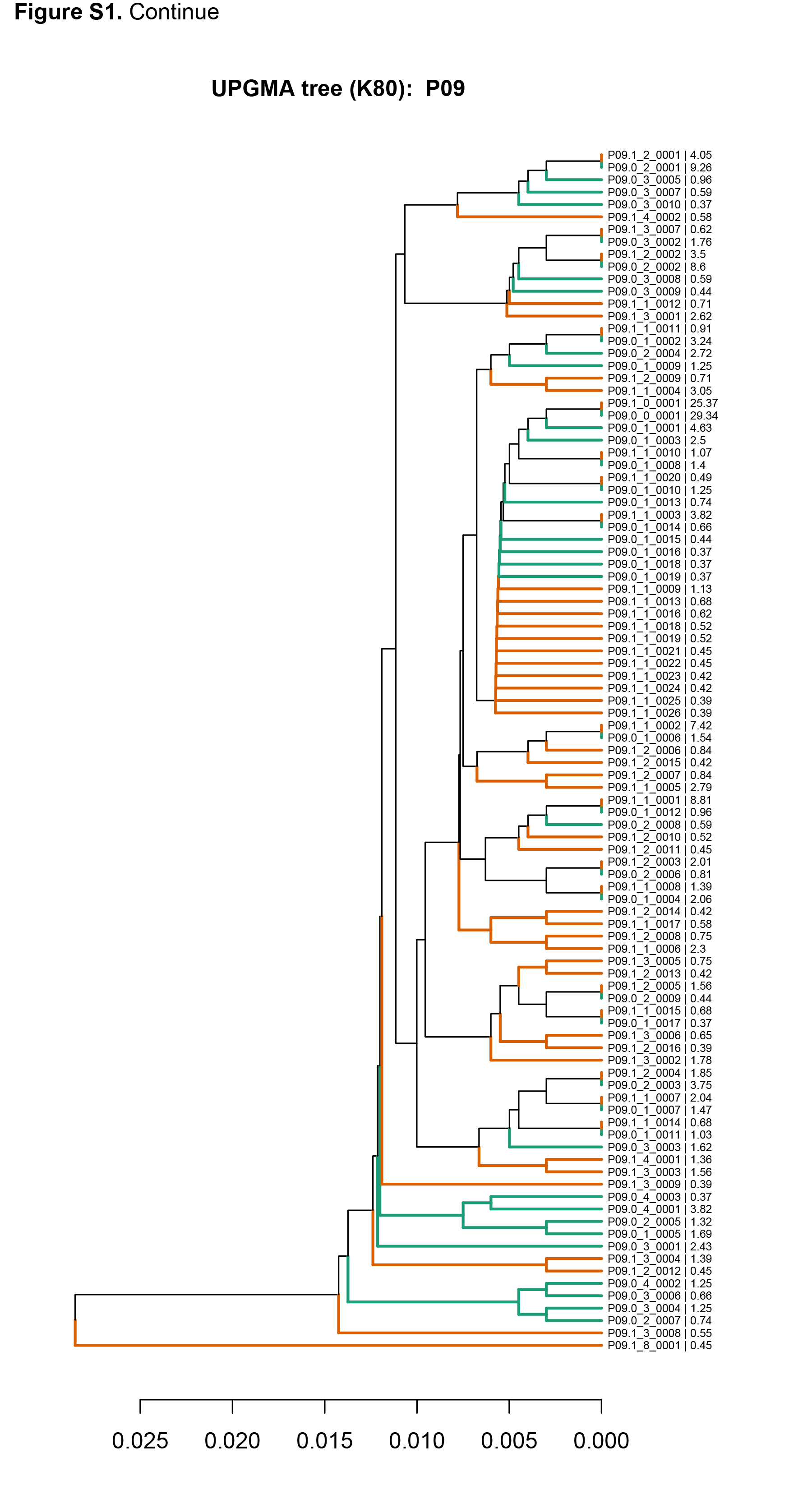

Supplement: Supplementary file 1 [file genes-12-01731-s001.zip › HplTreeS1.6.tif]

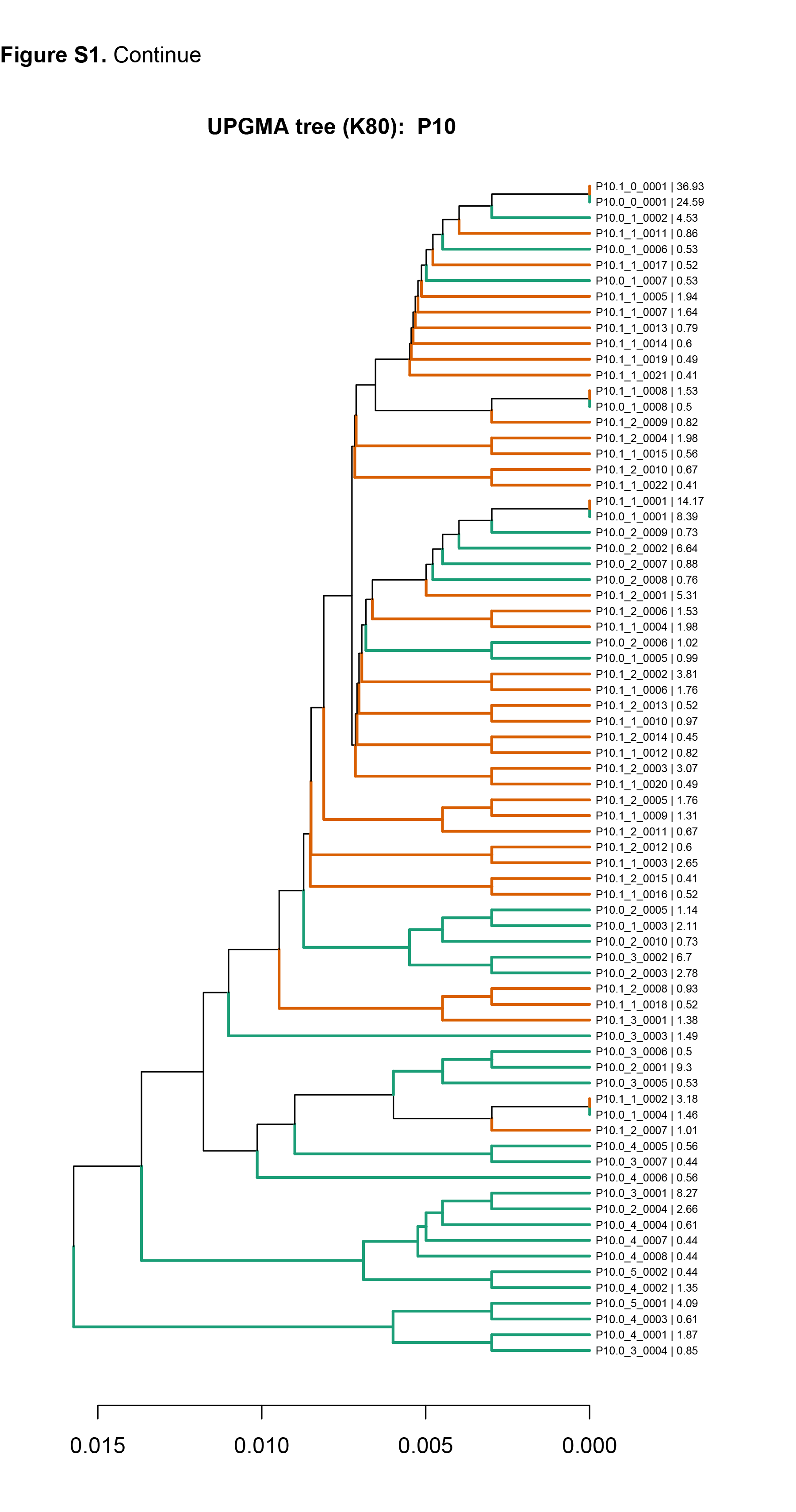

Supplement: Supplementary file 1 [file genes-12-01731-s001.zip › HplTreeS1.7.tif]
